# Mitigating Shrinkage in Superplasticizer-Free Natural Hydraulic Lime Grouts for Historic Masonry Conservation: Effects of Composition, Curing, and Expansion Agents

**DOI:** 10.3390/ma18163791

**Published:** 2025-08-13

**Authors:** Yang Wu, Shibing Dai

**Affiliations:** College of Architecture and Urban Planning, Tongji University, No. 1239 Siping Road, Shanghai 200092, China; wuyang5279@tongji.edu.cn

**Keywords:** historic masonry conservation, natural hydraulic lime, grout shrinkage, expansive agent, superplasticizer-free, mitigation strategies

## Abstract

Cracking is the most prevalent deterioration issue in historic masonry, and grouting represents one of the most effective intervention techniques. Superplasticizer-free Natural Hydraulic Lime (NHL) grout is recommended for heritage conservation due to its simple composition and compatibility with historic masonry in terms of strength, porosity, and other properties. However, grout shrinkage is frequently observed in practice, often leading to suboptimal reinforcement outcomes. This study focuses on the shrinkage characteristics of NHL grouts. Three sets of experiments were designed to investigate the influence: grout composition, expansive agents, and substrate properties. Using Taguchi’s method, an optimized combination of water, binder, and aggregate was identified. Shrinkage measurements after curing for 28 days demonstrated that calcium oxide (CaO)-based expansive agents was the best choice to compensate for NHL grout shrinkage. In addition, grouting simulation experiments evaluated suitable formulations for common masonry substrates and clarified the significant impact of substrate water absorption on the degree of shrinkage grout. For substrates with a capillary water absorption coefficient greater than 25 kg/m^2^ h^1/2^, the use of expansive agents should be strictly controlled. The findings can provide valuable insights for optimizing the grouting reinforcement of historic masonry structures and offer direct material design strategies for practical engineering applications.

## 1. Introduction

Masonry is a material composed of natural or manually manufactured units joined with fresh mortar, which constitute an important inventory of existing buildings in the world, from Egyptian civilization to the present day [1].

**Figure 1:**
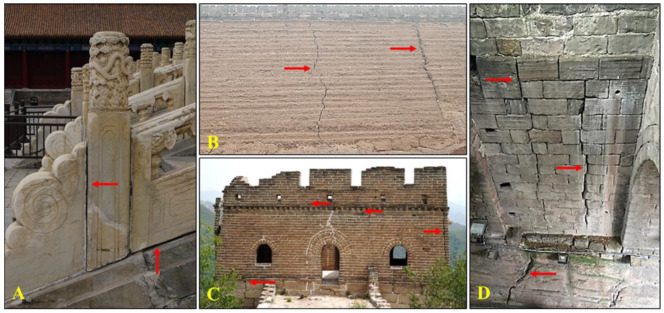
Cracks in the monuments and walls: (**A**) Marble balster in the Forbidden City; (**B**) the wall of Pingyao ancient city; (**C**) the Great Wall in Hebei; (**D**) the ancient city wall of Tongyuanmen in Chongqing.

Cracks and voids were common deterioration patterns in historic masonry. They occurred when the stress in the components exceeded its strength, either by man-made or natural factors, such as dead, live, wind, or seismic loads, foundation settlement, etc. [2]. Alternatively, it could be induced internally due to thermal movements, moisture changes, elastic deformation, chemical action, etc. In the masonry constructed by stones and bricks, most of the cracks developed because the bonding materials fade away (Figure 1A,D).

Fractures and unnoticeable tiny cracks are both signals of danger for the masonry. On the one hand, these cracks can be a conduit for water and contaminants to enter the interior of the masonry, where they undergo a series of complex reactions that accelerate the degradation [3]. On the other hand, tiny cracks on the surface indicate that there may be serious problems inside the masonry which have not yet been detected [4]. Therefore, once cracks were found in historical buildings, timely evaluation and appropriate intervention measures need to be taken [5].

Grouting is one of the techniques most widely used in the consolidation of such cracks or voids in the buildings [6,7]. The materials initially used for grouting were primarily natural clay, volcanic ash, or lime made from calcined calcium-containing stone [8,9]. After the invention of cement in 1824, it quickly gained widespread global adoption due to its high strength and adaptability. In the early 19th century, chemical grouting technology began to emerge, water glass, acrylic, polyurethane, and epoxy resin being used for building reinforcement. Currently, grouting materials tend to be composite, with advanced nanomaterials, graphene, as well as biomaterials being introduced and continuously optimized, significantly enhancing the performance of grouts [10,11]. However, considering compatibility with heritage buildings and the reversibility of intervention measures, lime-based materials have remarkable advantages in the restoration of historic masonry, especially the natural hydraulic lime (NHL) [12].

NHL was produced by the burning of argillaceous or siliceous limestones, with reduction to powder by slaking with or without grinding. It has the property of setting and hardening when mixed with water and by reaction with carbon dioxide from the air [13]. Years of research have verified that NHL has excellent properties such as moderate strength, good permeability, and compatibility with porous materials, in addition to its simple chemical composition, which does not introduce soluble salts [14]. In recent years, the majority of studies on NHL have focused on enhancing its strength and weather resistance by incorporating materials such as pozzolans, calcined clays, and fibers [15,16,17,18]. Some experiments have also investigated the positive effects of using ammonium and diethyl carbonates to accelerate the carbonation process of NHL materials [19,20]. However, critical properties for grouting applications, such as injectability and shrinkage, have not received sufficient attention despite lime-based materials exhibiting significantly higher shrinkage than cementitious systems [21].

**Figure 2:**
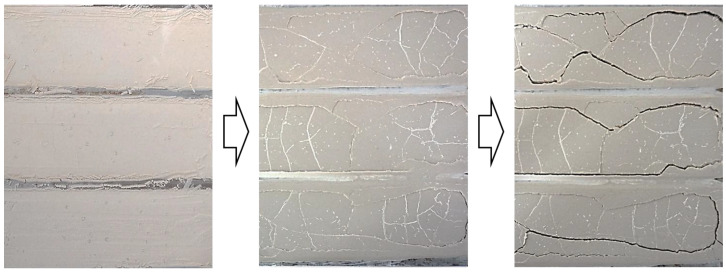
Cracks caused by shrinkage of the lime grouts.

Typically, grouts consist of three parts: aggregate, cementitious material, and water. In this case, water participates in the hydration reaction of the cementitious material as a reactant on the one hand, but more importantly serves as a medium to make the grouts fluid [22]. Within a short period of time after the grouting has been completed, the water in the grout will either be absorbed by the masonry or dissipated into the air by evaporation, both of which together result in significant volumetric shrinkage. Then, as the hydration reaction and carbonation reaction proceed, chemical shrinkage, autogenous shrinkage, and carbonation shrinkage occur simultaneously [23]. The above changes not only create small cracks within the hardened grouts, reducing the mechanical strength but also creates gaps at the interface between the grouts and the masonry, weakening the filling and bonding effect. As shown in Figure 2, shrinkage leads to fine fissures within the hardened grouts, and it break down into fragments along the fissures when attacked. So, it is no doubt that controlling shrinkage is a must.

Based on different mechanisms of action, there are primarily two main methods for reducing the drying shrinkage [23,24,25]. The most direct and effective approach involves introducing shrinkage-reducing admixtures (SRA), expansive additives (EA), or adjusting external curing conditions. These three techniques directly suppress the tensile stresses causing shrinkage by limiting the formation of liquid surfaces in capillaries or through the expansion of expansive agents. The second method involves enhancing the microstructure to offset drying shrinkage, which can be achieved by adding fibers, aggregates, mineral admixtures and other materials to the grouts. It should be noted that the addition of the above-mentioned admixtures can only mitigate shrinkage when the total water–binder ratio remains constant. But in the practice, it is first necessary to ensure the fluidity of the grouts. The addition of fine particles inevitably often increased the demand for water, so superplasticizers were used in conjunction. For the pure lime grouts, superplasticizers can reduce the w/b ratio while maintaining workability, thereby significantly reducing drying shrinkage [26,27,28,29].

However, these additives often introduce substances that are incompatible with historical bricks or stones, posing a potential threat to the fragile and precious cultural heritage. The most common are soluble salts, whose destructive effect on porous materials such as masonry is indisputable [30,31]. Fiber materials were excellent for mortar, but when added to grout, they may weaken its ability to penetrate tiny cracks. So, for the conservation of historical masonry, it is essential to develop grout without additives [32].

This paper focused on the simplest hydraulic lime grouts without superplasticizers. A series of experiments were conducted to investigate how the mix proportions, suitable expansive agents, and the properties of reinforced masonry affect the final shrinkage of the lime grouts. These findings will provide valuable reference for the use of superplasticizer-free hydraulic lime for masonry grouting or crack injecting in heritage conservation.

## 2. Materials and Method

**Figure 3:**
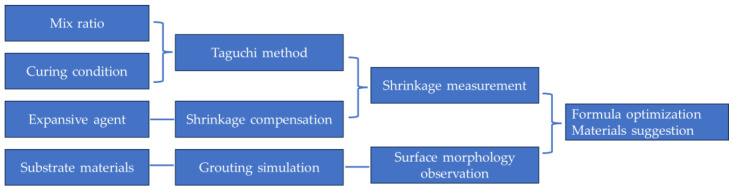
Flow chart of experimental program.

This study conducted experiments on four main factors that may affect the shrinkage rate of the grouts, namely the following: mix ratio, curing conditions, expansive agent, and the water absorption of the substrate materials. The flow chart of the experimental program is shown in Figure 3.

### 2.1. Shrinkage Experiment

#### 2.1.1. Raw Materials

In this study, marble powder and three types of natural hydraulic lime were used to produce the lime grouts. All of these materials are commercial products available in the market. Hydraulic lime named NHL5 and NHL2 were produced in Wiesloch in Germany by Hessler Kalkwerke Gmbh, while the NHL3.5 was produced in Dordogne in France by St.Astier. Both of them were bought from Zhejiang Desaibao Building Materials Co., Ltd. in Huzhou, China. The marble powder was produced by Changxing Qingsheng Calcium Industry Co., Ltd. in Huzhou, China. It was used as an aggregate in this study. The characteristics of the raw materials were obtained from the manufacturer and shown in Table 1.

It should be noted that three types of hydraulic lime were used in the investigation of the shrinkage properties of different types of hydraulic lime. Only NHL2 was used in the following experiment on the influence of factors such as aggregate ratio, water ratio, and curing conditions.

#### 2.1.2. Sample Preparation

In this study, the lime grouts were prepared at the laboratory with T = 20 ± 2 °C, RH = 60 ± 5%. Following the optimal mixing procedure described in [33], whole lime and marble powder were poured into the mixer cup together, while the water was added in two stages: 70% of the total amount added first, stirred for 30 s, and then the remaining 30% was added without stopping the machine. After all materials have been added, mix for 4 min at 2100 rpm.

As many factors affect the grout behavior and stability, the use of the Taguchi method can substantially reduce the number of tests. In this study, three control factors were chosen: percentage of the aggregate (A), w/b ratio (B), and the curing condition (C). For each factor, three control levels were considered, as shown in Table 2. According to this combination of three level factors, a standard L9 orthogonal array was used. After conducting the tests, the analysis of results was made using average values. In order to distinguish the impact of all various factors on the degree of shrinkage, range analysis was applied to the three factors mentioned before.

#### 2.1.3. Curing

**Figure 4:**
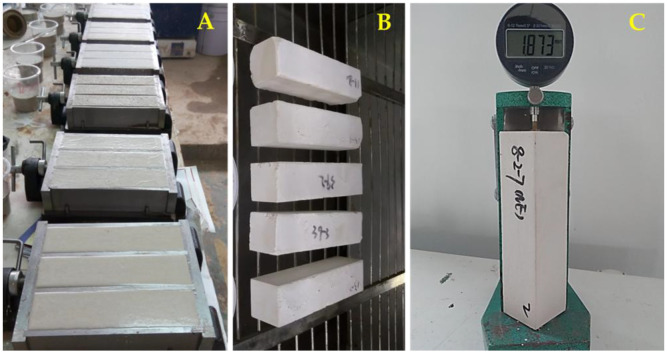
Sampling and shrinkage evaluation.

The freshly molded grouting samples were placed inside polyethylene bags for 3 days for initial curing (Figure 4A), then demolded and divided into three groups, each group corresponding to a distinct curing condition [34]. The curing conditions employed were: C1—laboratory-controlled humid curing, with temperature (T) = 10 ± 2 °C and relative humidity (RH) = 35 ± 5%; C2—laboratory controlled standard curing, where the grouts were placed in T = 20 ± 2 °C and RH = 65 ± 5%; C3—laboratory-controlled humid curing, with temperature (T) = 30 ± 2 °C and relative humidity (RH) = 95 ± 5% (Figure 4B).

#### 2.1.4. Shrinkage Evaluation

The length of the samples was measured and recorded by the length instrument after demolded (Figure 4C). According to the test setup, the nine groups of samples were placed under the corresponding conditions, and the length of the specimens was measured at the age of 3, 5, 7, 14, 21, 28, 35, 42, 49, 56, and 90 days by the means referring to the national standard JCJ/T 70-2009 [35]. Each group consists of three samples, each of which was tested three times, with the arithmetic mean serving as the final result. Shrinkage rate of the samples is calculated according to Equation (1).

(1) $$\varepsilon_{t}=\frac{L_{0}-(L_{s}+C_{t})}{L_{0}}\times 100\%$$

where $\varepsilon_{t}$ is the shrinkage of samples at different ages, $L_{s}$ is the length of the standard bar (there is 158.04 mm), $C_{t}$ is the reading value of the instrument, and $L_{0}$ is the initial value of sample length (there is 160 mm).

### 2.2. Shrinkage Compensation Test

**Figure 5:**
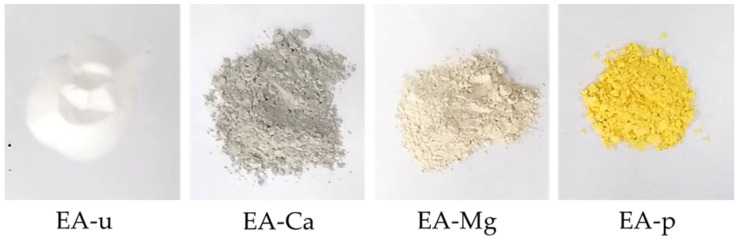
Four expansive agents used in this study.

In many cases, shrinkage of the grouts cannot be avoided just by adjusting the composition, materials with expansive properties are added to compensate for the shrinkage. In this study, four expansive agents named EA-ca, EA-Mg, EA-u, and EA-p have been tested; their main chemical content and properties were obtained from manufacturer and shown in Table 3, and the particle morphology and surface color are shown in Figure 5.

### 2.3. Grouting Simulation

Masonry was made up of a variety of materials such as brick, stone, earth, and so on, each of which has a different level of water absorption, thus affecting the degree of shrinkage when grouting finished.

Four substrates—sandstone, green clay brick, white marble, and the plastic board— were used to simulate historic masonry that needs to be grouted for repairs. The capillary water absorption coefficient of these four materials was tested according to the WW/T 0063-2015 [36] and shown in Table 4. They were cut into rectangles of the same size and placed in the bottom of the box, and then the NHL grouts with 6% Cao expansive agent were injected. After being cured at room temperature in the laboratory for 90 days, the surface morphology was observed and recorded.

## 3. Results and Discussion

### 3.1. Forms of Shrinkage Cracks

**Figure 6:**
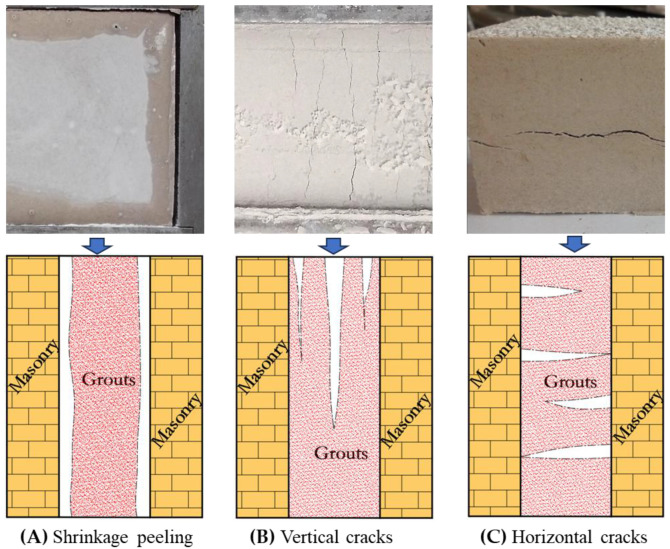
Picture and schematic diagram of cracks in the grouts.

After the lime grouts were poured into the molds, cracks perpendicular to the test prism were observed on the surface, as a series of reactions took place (Figure 6B). Three days later, the severely shrunken samples had separated from the inner wall of the mold, revealing significant gaps (Figure 6A). Upon demolding, internal horizontal cracks parallel to the mold baseplate were additionally identified in some grout samples (Figure 6C).

### 3.2. Parameters Related to the Shrinkage of Lime Grouts

#### 3.2.1. Type of NHL

**Figure 7:**
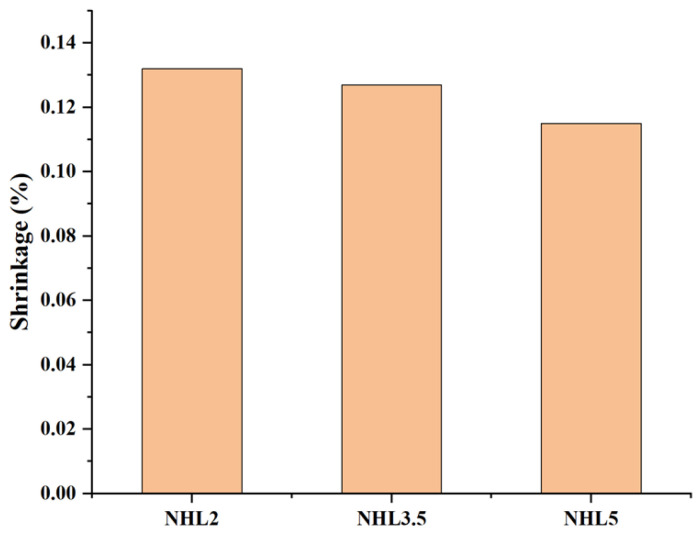
Shrinkage of different type of hydraulic lime.

Figure 7 shows the shrinkage of different type of NHL grouts, which were cured under the room temperature for 90 days. NHL2, NHL3.5, and NHL5 are three subtypes of natural hydraulic limes, and the numbers represent the compressive strength after 28 days under standard curing conditions. In this study, the grout prepared with the three different kinds of lime showed less difference in shrinkage, with shrinkage ranging from 0.12% to 0.13%. The lowest shrinkage was observed for NHL5 grout and the highest for NHL2. Combined with the higher content of both silica and aluminum trioxide oxides shown in Table 1, which indicates that NHL5 has a higher hydraulicity activity, enables it to react quickly with water, solidify, and generate strength, preventing the water in the grout from drying out and limiting subsequent shrinkage.

#### 3.2.2. Curing Age

**Figure 8:**
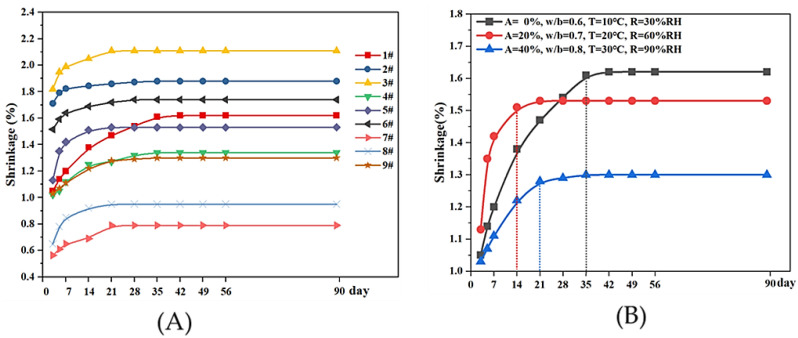
Shrinkage of the grouts over the age. (**A**): All grout samples showed a trend of shrinkage slowing down over time. (**B**): Time required for grout shrinkage to reach a stable state under different conditions.

Through the observation of the nine groups for a period of 90 days, results show that with the extension of curing age, the shrinkage of all the samples increased gradually (Figure 8).

As the curing time increases, the rate of shrinkage of the grouts sample changes from fast to slow until it stabilizes. Under laboratory curing conditions, the turning point usually occurs on the 14th day, test data show that the shrinkage rate on the 14th day can reach 95% of the final value; in the colder and drier environments, this time is doubled, the shrinkage of the grout specimens reached its maximum value at about the 5th week. In the hot and humid environments, this experiment observed that the shrinkage of the samples reached a steady state after the 3rd week.

#### 3.2.3. Aggregate and Water/Binder Ratio

**Figure 9:**
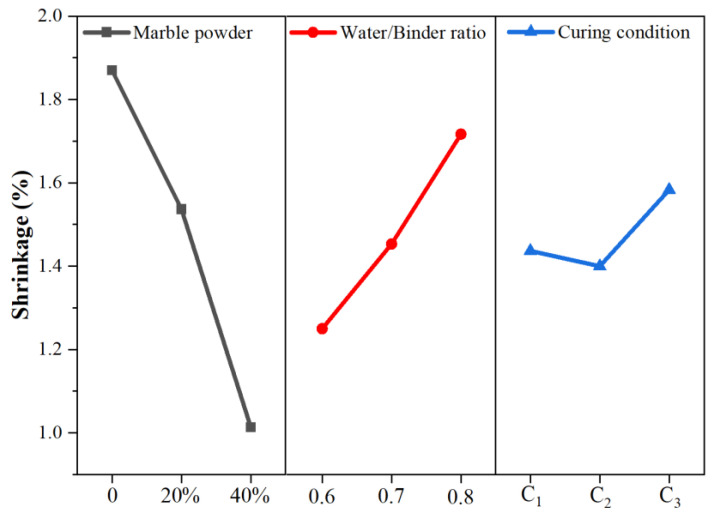
Influence of factor effects on shrinkage.

**Figure 10:**
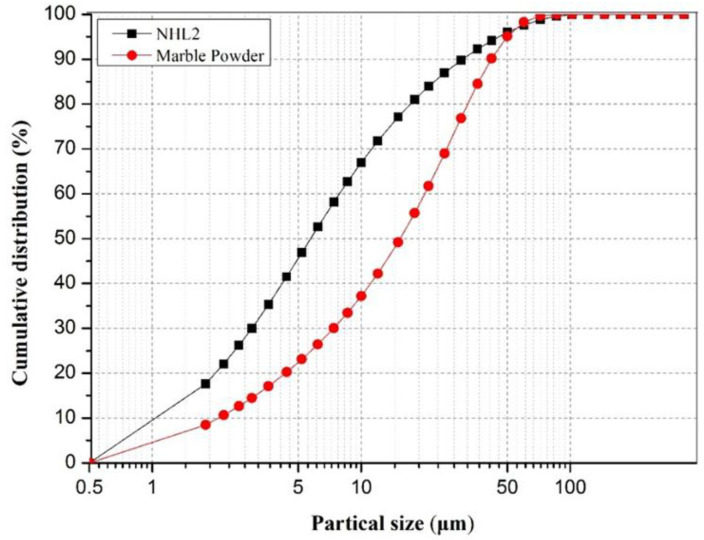
Particle size distribution of the NHL2 and marble powder.

The shrinkage of the sample for 90 days was taken as the final shrinkage value, and the results of the orthogonal test were analyzed by the extreme value method. In Table 5 and Figure 9, we can see the difference in the factor A (R_A_) was the largest, which is 0.86, while the R_b_ and R_c_ were 0.26 and 0.18, respectively. It means that the grouts’ shrinkage gradually decreased with the addition of marble powder. Pure lime grouts exhibit significant shrinkage, while with the addition of 40% marble powder as an aggregate, the shrinkage reduced by 40% accordingly, such significant change is mainly related to two factors, firstly, marble powder dispersed into the grouts creates a stronger framework, which can resist the volume shrinkage. In addition, considering the particle size distribution of NHL and marble powder in Figure 10, More than 70% percent of the lime particles have a size of less than 10 um, compared to only 30% percent of the marble powder. This means that stone powder has a lower specific surface area than lime, and under the premise of satisfying the workability of the grouts, the water requirement is reduced after the addition of aggregates, and the subsequent shrinkage is naturally reduced.

The w/b ratio has a more direct effect on the grout’s shrinkage, as the ratio increased from 0.6 to 0.8, the shrinkage rise by 40%. Due to the low content of the hydraulic component in NHL2, the water requirement for the hydration reaction is very small, and the excess water in the grouts merely ensures fluidity. Therefore, the higher the w/b ratio, the more free water there is in the slurry, and, ultimately, the greater the shrinkage caused by the loss of water.

Therefore, from the point of view of reducing the shrinkage, under the premise of ensuring the constructability, the aggregate can be added appropriately to reduce the water–binder ratio. In this experiment, the optimal ratio combination was A_3_B_1_C_2_, which means using 40% marble powder as aggregate, controlling the w/b ratio to 0.6, and curing at the environment with 20 °C and 60% RH.

#### 3.2.4. Curing Condition

The effect of curing conditions on the shrinkage of the sample is mainly reflected in the environmental factors, which will affect the evaporation rate of free water in the grouts. In this study, three different nature conditions were simulated by the test instruments, they were low temperature and dry condition (10 °C, RH30%), room temperature and moderate humid condition (20 °C, RH60%), and the high temperature and high humid condition (30 °C, RH90%). As shown in Table 5 and Figure 9, we can see that the samples with smallest shrinkage were the one cured at room temperature and humidity conditions, followed by the samples in the low temperature drying condition, and the largest one was the sample cured in the high temperature and high humidity condition. Some reports have shown that the hydration reaction is more adequate in the condition of 20 °C, RH60% [34]. So, it was possible that the adequate hydration reaction led to a decreased shrinkage too. It should be noted that the difference between them is small, that is, the curing environment has very little effect on the final degree of shrinkage of the grouts and, conversely, has a more pronounced effect on the rate of shrinkage (Figure 9).

### 3.3. Shrinkage Compensation by Expansive Agent

EA-ca was the CaO expansive agent, wherein the main component is calcium oxide; it can react with water to produce calcium hydroxide, resulting in the volume being increased by approximately 1.98 times [37]. Similarly, the magnesium oxide in the MgO expander (EA-Mg) can convert to magnesium hydroxide crystals, which fill the pores of the solid material, leading to macroscopic volume expansion. The main components of UEA expansive agent (EA-u) are alumina, gypsum, and sulphoaluminate, which can expansion by produce ettringite (Aft) through a series of complex hydration reactions. Plasticity Expanders (EA-p) in this study are obtained by calcining bauxite, which will release nitrogen gas when added in the alkaline environment of lime slurry, causing rapid expansion of the grout’s volume [38].

Many studies have discussed the expansion effect of the above four expanders in cement and concrete mortar [39,40], while the use in lime grouts was unknown. In this study, natural hydraulic lime, NHL2, was used as a raw material to evaluate the effect of shrinkage compensation about four different expansive agents on hydraulic lime grouts.

**Figure 11:**
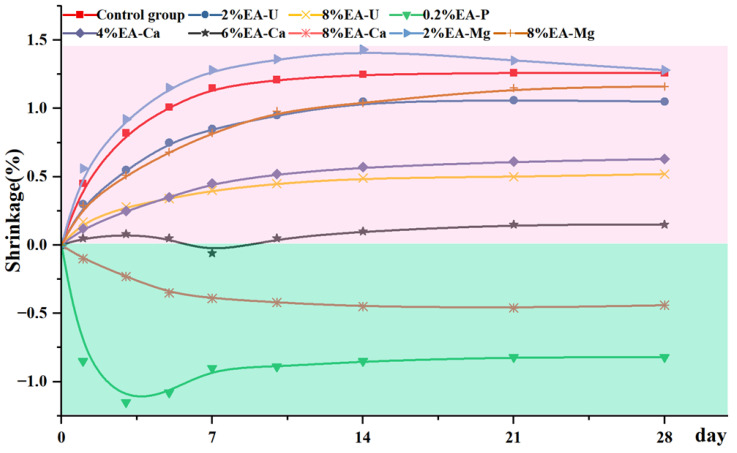
Shrinkage of lime grouts with expansive agent.

**Figure 12:**
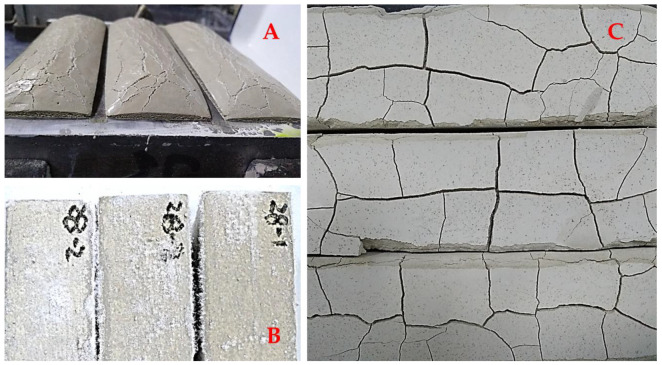
Inappropriate use of expansive agent ((**A**) 0.2%plastic expansive agent causing excessive expansion; (**B**) Soluble salt caused by Sulfoaluminate expansive agent; (**C**) Excessive calcium oxide expansive agent leads to fragmentation of the hardened grouts).

Figure 11 has shown the results of the shrinkage test of the lime grouts with the addition of the different types of expanders. The area with red background indicated that the expansive agent has not yet fully compensated for the shrinkage of the grouts, while the green background area indicated expanded significantly. Twenty-eight days of continuous monitoring results show that all four types of expansive agents can compensate for the shrinkage more or less. Among them, the plastic expansive agent being the most effective, with a dosage of only 0.2%, resulting in an expansion of more than 1%. Because of the huge expansion, the thickness of the hardened grouts even exceeded the mold edge (Figure 12A).

EA-Mg had the least compensatory effect, the shrinkage of the grouts still reaching 0.9% at 8% dosage, and in comparison, the magnesium oxide expander had a later onset of action, grouts expansion exhibited after approximately 14 days of curing. Calcium oxide expanders and UEA expanders have similar effects, but it was observed that many salt crystals appeared on the surface of the samples after dosing with UEA expanders, which makes it inappropriate for heritage masonry conservation (Figure 12B). The calcium oxide expander is most effective, as the calcium hydrate is more compatible with historic substrate. However, its dosage needs to be strictly controlled. In this experiment, 6% is the optimum, and when it reached 8%, the hardened lime grouts break down into small fragments (Figure 12C).

### 3.4. Effect of Substrate on Shrinkage

Masonry was made up of a variety of materials such as brick, stone, earth, and so on; each of which has a different level of water absorption, thus affecting the degree of shrinkage when grouting finished.

**Figure 13:**
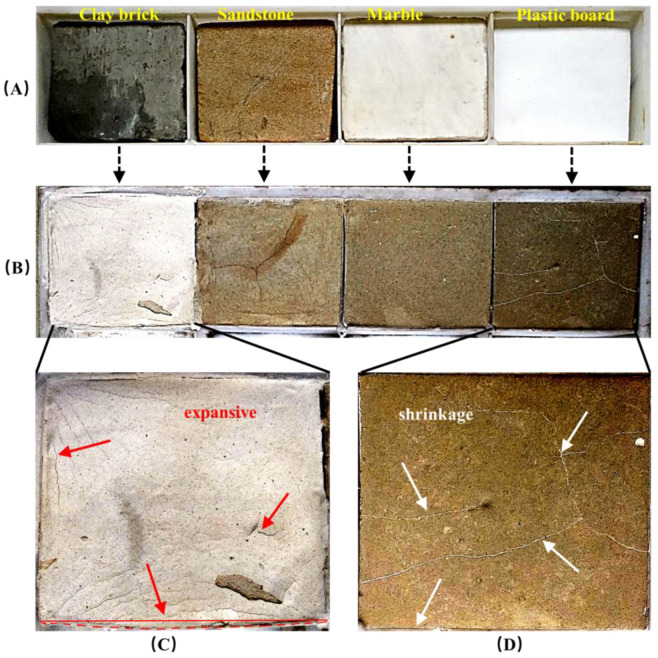
Comparison before and after grouting. (**A**): Four substrate materials used for grouting simulation; (**B**) After grouting; (**C**) Cracks caused by grout expansive; (**D**) Cracks caused by grout shrinkage.

As shown in Figure 13A, four substrates—sandstone, green clay brick, white marble, and the plastic board—were used to simulate historic masonry that needs to be grouted. The capillary water absorption coefficient of these four materials was shown in Table 4. They were cut into rectangles of the same size and placed in the bottom of the box, and then the grouts based on NHL2 with 6% Cao expansive agent were injected. After being cured at room temperature in the laboratory for 90 days, the surface morphology was observed and recorded (Figure 13B).

Samples based on green clay brick showed significant swelling and increased volume of the hardened grout. The red solid line in Figure 13C was the original boundary of box, and the actual boundary after swelling is the red dashed line—the obvious change was due to the expansion of the grouts. A similar fissure caused by expansion also appeared on the surface of the sandstone in Figure 13B. However, for the sample with plastic board as the substrate, multiple cracks appeared on the surface as a result of the shrinkage, especially in the area between the hardened grouts and the mold edge, wherein the grouts have already separated from the inner surface of the mold (Figure 13D).

Combined with the effect of w/b ratio on grouts shrinkage as described in the second part in this paper, the main reason for the different shrinkage behavior of the same grouting material is the different water absorption of the substrate. For materials with good water absorption, within a short period of time after the lime grouts come into contact with the substrate, a large amount of free water in the grout is sucked away, then the w/b ratio decreases correspondingly and, therefore, the shrinkage reduced, or even expanded.

On the contrary, for poorly absorbent materials, such as marble (Table 4), the free water content in the grouts hardly decrease before and after grouting, so its shrinkage properties almost unchanged. If at this time, the prepared grouts do not contain enough expansive agent, they will eventually show significant shrinkage.

Through the grouting simulation experiments in this study, it can be determined that NHL grout mixed with 6% calcium oxide expansive agent is suitable for reinforcing marble cracks with water absorption coefficients between 2 and 5 kg/m^2^ h^1/2^. But for other types of materials, it was necessary to adjust the formulation or grouting technique.

## 4. Conclusions and Outlook

Natural hydraulic Hydraulic lime was widely used in architectural heritage conservation due to its good compatibility. However, when used as grouting material, the filling and bonding effect was often unsatisfactory due to the shrinkage. In this study, a series of experiments were carried out around the shrinkage properties of lime grout, yielded the following conclusions:

(1) Shrinkage of lime grouts was mainly affected by aggregate and w/b ratio. When the w/b increased from 0.6 to 0.8, the shrinkage rises by 40%; however, while with the addition of 40% marble powder as an aggregate, the shrinkage reduced by 40%.
(2) The three natural hydraulic limes, NHL2, NHL3.5, and NHL5, showed almost identical in terms of shrinkage properties, but NHL5 has lowest shrinkage performance under same conditions
(3) Under the standard curing conditions (T = 20 ± 2 °C, RH = 65 ± 5%), the shrinkage of NHL grout stabilizes within 14 days, but this time will be delayed to 3 even 5 weeks if the curing environment is hot and humid, or if it was dry and cold.
(4) Magnesium oxide expanders were slow-acting, making it difficult to determine the optimum dosage in a short time; only 0.2% of plastic expanders can lead to significant expansion, making it difficult to control in practice. UEA expanders are also not recommended because they bring soluble salts.
(5) Calcium oxide expansive agent was recommended, with calcium hydroxide as the reaction product, which was the same as that of the lime grout, and the 5–10% dosage was also easy to mix during practical application.
(6) Special attention needs to be paid to the fact that the water absorption of the masonry being repaired will significantly affect the final shrinkage or expansion. For the materials with high water absorption, such as sandstone and old bricks, whose capillary water absorption coefficient exceed 25 kg/m^2^ h^1/2^, it is recommended to control shrinkage by optimizing the proportion of grouts rather than using expansive agents. For weakly absorbent substrate materials such as granite or marble, expansive agents should be added appropriately.

Overall, lime grouts without superplasticizer hold great promise for heritage conservation. Optimizing the composition of the grouts according to the strength and water absorption characteristics of the historic masonry is a prerequisite for the effectiveness of the restoration. In subsequent work, a lot of experiments still need to be conducted to obtain enough data and develop a predictive model linking grout shrinkage to formulation parameters, curing conditions, and substrate properties.

## Figures and Tables

**Table 1:** Chemical composition (oxide wt%) and physical properties of raw materials.

Materials	Oxides Composition (wt%)					Apparent Density (g/cm^3^)	Surface Color
	Al_2_O_3_	Fe_2_O_3_	SiO_2_	CaO	MgO		
NHL2	1.10	0.55	11.20	62.60	1.44	2.21	ash gray
NHL3.5	1.59	0.58	18.89	55.95	1.90	2.29	ash gray
NHL5	1.62	0.58	19.98	55.60	1.02	2.46	ash gray
Marble powder	6.53	0.29	4.85	80.61	0.06	2.72	matte white

**Table 2:** Orthogonal array L9 with factors levels assignment for the tests.

	Factors		
	A (Marble Powder)	B (Water/Binder Ratio)	C (Curing Condition)
1	1 (0%)	1 (0.6)	1 (C_1_ = 10 °C, 35% RH)
2	1 (0%)	2 (0.7)	3 (C_3_ = 30 °C, 95% RH)
3	1 (0%)	3 (0.8)	2 (C_2_ = 20 °C, 65% RH)
4	2 (20%)	1 (0.6)	3 (C_3_ = 30 °C, 95% RH)
5	2 (20%)	2 (0.7)	2 (C_2_ = 20 °C, 65% RH)
6	2 (20%)	3 (0.8)	1 (C_1_ = 10 °C, 35% RH)
7	3 (40%)	1 (0.6)	2 (C_2_ = 20 °C, 65% RH)
8	3 (40%)	2 (0.7)	1 (C_1_ = 10 °C, 35% RH)
9	3 (40%)	3 (0.8)	3 (C_3_ = 30 °C, 95% RH)

**Table 3:** Characteristic of expansive agent.

	Main Content (%)					Specific Surface Area (m^2^/kg)	Surface Color
	CaO	MgO	SiO_2_	Fe_2_O_3_	Al_2_O_3_		
EA-Ca	74.5	4.3	3.3	3.1	2.5	345	dark gray
EA-Mg	3.5	82.5	6.3	0.7	0.9	320	light brown
EA-u	11.3	2.5	40.1	4.7	14.9	270	white
EA-p	27.5	22.5	14.5	13.2	3.4	315	faint yellow

**Table 4:** Properties of the substrate materials used in the test.

Substrate Material	Total Porosity (%)	Capillary Water Absorption Coefficient (kg/m^2^ h^1/2^)
Sandstone	12.20	8.40
Green clay brick	28.90	18.70
White marble	0.56	0.08
Plastic board	0.00	0.00

**Table 5:** Results of the Extreme value method analyzed.

	Factor			Result
	A	B	C	Shrinkage (%)
1	1(0)	1(0.6)	1(C1)	1.62
2	1	2(0.7)	2(C2)	1.88
3	1	3(0.8)	3(C3)	2.11
4	2(20)	1	3	1.34
5	2	2	2	1.53
6	2	3	1	1.74
7	3(40)	1	2	0.79
8	3	2	1	0.95
9	3	3	3	1.30
K_1_	5.61	3.75	4.31	Σ = 13.26
K_2_	4.61	4.36	4.2	
K_3_	3.04	5.15	4.75	
${\overset{-}{k}}_{1}$	1.87	1.25	1.44	
${\overset{-}{k}}_{2}$	1.54	1.45	1.40	
${\overset{-}{k}}_{3}$	1.01	1.72	1.58	
Excellent level	A_3_	B_1_	C_2_	
R	0.86	0.26	0.18	

## Data Availability

The original contributions presented in this study are included in the article. Further inquiries can be directed to the corresponding author.
